# Antiparasitic and Antibacterial Functionality of Essential Oils: An Alternative Approach for Sustainable Aquaculture

**DOI:** 10.3390/pathogens10020185

**Published:** 2021-02-09

**Authors:** Mahmoud A. O. Dawood, Mohammed F. El Basuini, Amr I. Zaineldin, Sevdan Yilmaz, Md. Tawheed Hasan, Ehsan Ahmadifar, Amel M. El Asely, Hany M. R. Abdel-Latif, Mahmoud Alagawany, Nermeen M. Abu-Elala, Hien Van Doan, Hani Sewilam

**Affiliations:** 1 Animal Production Department, Faculty of Agriculture, Kafrelsheikh University, Kafr El-Sheikh 33516, Egypt; 2 The Center for Applied Research on the Environment and Sustainability, The American University in Cairo, Cairo 11835, Egypt; 3 Faculty of Desert Agriculture, King Salman International University, South Sinai 46618, Egypt; m_fouad_islam@yahoo.com; 4 Department of Animal Production, Faculty of Agriculture, Tanta Uni-versity, Tanta 31527, Egypt; 5 Animal Health Research Institute (AHRI-DOKI), Agriculture Research Center, Kafrelsheikh 33511, Egypt; dramrzaineldin@gmail.com; 6 Department of Aquaculture, Faculty of Marine Sciences and Technology, Canakkale Onsekiz Mart University, Canakkale 17100, Turkey; sevdanyilmaz@comu.edu.tr; 7 Department of Aquaculture, Sylhet Agricultural University, Sylhet 3100, Bangladesh; tawheed7788@yahoo.com; 8 Department of Fisheries, Faculty of Natural Resources, University of Zabol, Zabol 98615-538, Iran; ehsanahmadifar@gmail.com; 9 Department of Aquatic Animals Diseases and Management, Faculty of Veterinary Medicine, Benha University, Benha 13511, Egypt; amlvet@yahoo.com; 10 Department of Poultry and Fish Diseases, Faculty of Veterinary Medicine, Alexandria University, Alexandria 21500, Egypt; hmhany@alexu.edu.eg; 11 Poultry Department, Faculty of Agriculture, Zagazig University, Zagazig 44511, Egypt; dr.mahmoud.alagwany@gmail.com; 12 Department of Aquatic Animal Medicine and Management, Faculty of Veterinary Medicine, Cairo University, Giza 12211, Egypt; nermeen_abuelala@cu.edu.eg; 13 Department of Animal and Aquatic Sciences, Faculty of Agriculture, Chiang Mai University, Chiang Mai 50200, Thailand; 14 Innoviative Agriculture Research Center, Faculty of Agriculture, Chiang Mai University, Chiang Mai 50200, Thailand; 15 Department of Engineering Hydrology, RWTH Aachen University, 52078 Aachen, Germany

**Keywords:** essential oils, parasite, bacteria, aquaculture, immune enhancer, medicinal plants

## Abstract

Using synthetic antibiotics/chemicals for infectious bacterial pathogens and parasitic disease control causes beneficial microbial killing, produces multi-drug resistant pathogens, and residual antibiotic impacts in humans are the major threats to aquaculture sustainability. Applications of herbal products to combat microbial and parasitic diseases are considered as alternative approaches for sustainable aquaculture. Essential oils (EOs) are the secondary metabolites of medicinal plants that possess bioactive compounds like terpens, terpenoids, phenylpropenes, and isothiocyanates with synergistic relationship among these compounds. The hydrophobic compounds of EOs can penetrate the bacterial and parasitic cells and cause cell deformities and organelles dysfunctions. Dietary supplementation of EOs also modulate growth, immunity, and infectious disease resistance in aquatic organisms. Published research reports also demonstrated EOs effectiveness against *Ichthyophthirius multifiliis*, *Gyrodactylus* sp., *Euclinostomum heterostomum*, and other parasites both in vivo and in vitro. Moreover, different infectious fish pathogenic bacteria like *Aeromonas salmonicida*, *Vibrio harveyi*, and *Streptococcus agalactiae* destruction was confirmed by plant originated EOs. However, no research was conducted to confirm the mechanism of action or pathway identification of EOs to combat aquatic parasites and disease-causing microbes. This review aims to explore the effectiveness of EOs against fish parasites and pathogenic bacteria as an environment-friendly phytotherapeutic in the aquaculture industry. Moreover, research gaps and future approaches to use EOs for sustainable aquaculture practice are also postulated.

## 1. Introduction

Farming of aquatic plants and animals is generally known as aquaculture, and the annual growth of this rapidly expanding food industry is 4.5%, accounting for a value of 243.26 billion USD [1] to meet up the protein demand of ever increasing world population. This important industry is also generating jobs, income, and providing 50% of global fish consumption [2,3]. Due to the increase of consumer demand, aquaculture technique has been shifted from extensive to super-intensive; intensification of aquaculture needs a higher amount of artificial feed supply, water treatment and reuse, and high stocking density resulting in aquatic environmental degradation [4,5,6]. Mounting of stress and quality deterioration of living environment increases the activity and virulence of infectious and opportunistic microbial pathogens [7], decrease immunity and immune-related gene transcription of aquatic animals [8], and elevate uni and multicellular parasitic infestation [9]; finally, initiate infectious diseases outbreak along with the death of cultured species. Gonzales, et al. [10] reported global aquaculture loss of 1.05 to 9.58 billion USD/year due to infectious diseases and parasitic attacks.

To eliminate diseases and parasitic attacks in the aquaculture industry, different synthetic antibiotics, chemical drugs, vaccines, and chemotherapeutics are being used at high rates from year after year [11,12]. Using of these chemical substances cause mass killing of beneficial aquatic bacteria [13], produce multi-drugs resistant pathogens [14], and leaving residues in fish which can be transmitted to human [15,16]. These problems are the most concerning aquaculture sustainability [17,18], and infectious diseases and parasitic infestation treatment with natural substances/compounds are the demanding sustainable aquaculture features [19].

The use of medicinal plants and their derivatives in aquaculture is increasing day by day all over the world because of having biodegradable properties [20,21,22,23,24], availability and ease to cultivate, and do not accumulate in animal tissues as a residue [25,26]. Essential oils (EOs) are the secondary metabolites of medicinal plants and possess bioactive properties to be used as a phytotherapeutic agent for sustainable aquaculture [27,28]. Terpens, terpenoids, phenylpropenes, and isothiocyanates are the key chemical groups identified in EOs [29]. EOs mainly penetrate and act upon the membrane and cytoplasm of bacteria to inhibit their action mechanisms by altering cell morphology and organelles deformities [30,31]. Generally, Gram-positive bacteria are more sensitive to EOs than Gram-negative due to lipoteichoic acids in cell membranes that might facilitate the penetration of EOs hydrophobic compounds [32]. According to Carson, et al. [33], EO comprises different compounds that have no specific cellular target in parasites. Monoterpenes α-pinene and sabinene of EOs have proved mentionable antiprotozoal activity. Moreover, synergistic effects of different compounds in EOs are another key feature that showed a higher mode of action relative to individual compounds. EOs cause leakage of potassium ions and cytoplasmic content of parasitic cells due to hydrophobicity and cell permeability, which cause cell morphology alteration and cessation of parasitic activity [34]. Staining with fluorocromes SYBR-14 and propidium iodide confirmand the plasma membrane damage in *Ichthyophthirius multifiliis* by the action of *Varronia curassavica* derived EOs [35].

Different microbial and parasitic diseases are the major threats to the aquaculture industry. Application of nanoemulsions EOs or other herbal products to combat microbial [36,37] and parasitic [9,25] diseases is considered a new alternative approach for sustainable aquaculture. Extensive research activities were performed for the identification and characterization of EOs effects for the fish and shellfish preservation and shelf life elongation [38,39], modulation of growth, immunity, and infectious disease resistance in commercially cultured fish species [35,40,41], against different pathogenic microbial activity [42,43] and destruction and retardation of fish parasitic activity [9,10]. In the fisheries and aquaculture sector, EOs act as a natural preservative [44], stress-reducing agent [45], herbal anesthetics [46], and oregano herb and medicinal plant as immunomodulators [26] and immunostimulants [47]. However, no study was conducted to identify EOs antiparasitic and antimicrobial properties for sustainable aquaculture.

Although natural EOs have enough potential for sustainable aquaculture, EOs have high volatility and can be decomposed by exposure to heat, humidity, light, and oxygen to lose effectiveness [48]. Application to the EOs in their oil form render it subjected to degradation during processing, storage, and handling [49]. The use of nano-encapsulated EOs becomes a promising trend in the field of EOs applications [50], especially in the aquaculture sectors [51], protecting the volatilization, low stability, low solubility in water, and associated problems of using EOs [52]. Nanoemulsion technology is currently solving the effectiveness disruption problems of EOs in aquaculture. This technology also protects EOs from the digestive enzyme’s actions in the intestine.

The main focus of this article is to identify EOs antimicrobial and antiparasitic properties that can be used for sustainable aquaculture practices. Moreover, EOs effects for aquaculture species growth, immunomodulation, and infection resistances were also postulated. In addition, research gaps and tentative future research activities are also mentioned to effectively use EOs in sustainable fish culture. 

## 2. EOs as Growth, Immunity, and Disease Resistance Enhancer

Several studies have been conducted to identify EOs growth and immunity elevation property; however, no specific research was conducted to identify the action mechanism of EOs for the alteration of these properties [28,53,54,55]. Jang, et al. [56] mentioned the possible reason for growth and feed utilization parameters modulation by EOs is due to elevation of digestive enzymes in the intestines. Moreover, EOs increased the appetite of aquaculture species [57] may be another reason. Antioxidant activity increased due to aromatic rings and the position of hydroxyl ion in EOs [58]. Modulation of the intestinal microbiome by EOs can be considered one of the possible reasons for the modulation of immune-related genes [59]. Significantly, phenolic compounds like thymol and carvacrol modulate innate immunity through two possible ways i) direct action on host tissue ii) influence on the intestinal microbial community [60].

A 60-day experiment was conducted with dietary supplementation with bitter lemon (*Citrus limon*) [61], and sweet orange peels (*C. sinensis*) [62] originated EOs in Mozambique tilapia (*Oreochromis mossambicus*). In both cases, EOs elevated innate immune parameters (NBT, WBCs, lysozyme, and myeloperoxidase activity) and decreased serum/blood glucose, cholesterol, and triglycerides. *C. limon* and *C. sinensis* EOs administrated tilapia demonstrated resistance against *Streptococcus iniae* and *Edwardsiella tarda*, respectively. In addition, a similar type of immunomodulation and infection protection of tilapia were also found after *C. limon* peel EOs supplementation at (1, 2, 5, and 8%) in *Labeo victorianus* for 28 days [63]. However, growth (WG% and SGR) and feed conversion ratio (FCR) modulation in the former study remained unchanged but in the latter two experiments increased significantly (Table 1). The authors claim active compound of EOs (limonene) concentration in the former experiment was 54.4%, whereas later studies were 94.74 and 81.40, respectively, may be the causal factors of these differences. In Nile tilapia (*O. niloticus*), lemongrass (*Cymbopogon citratus*) and geranium (*Pelargonium graveolens*) [40], and Oregano (*Origanum vulgare*) [64], supplementation increased growth and feed utilization, and resistance against the action of *Aeromonas hydrophila* and *Vibrio alginolyticus*, respectively. *C. citratus* and *P. graveolens* supplemented fishes not only improved immunity but also decreased the concentration levels of intestinal coliforms, *Escherichia coli*, and *Aeromonas* spp. Moreover, origanum EOs (1 g/kg) improved immunity and vibriosis protection in *Tilapia zillii* [65]. 

Eight weeks feeding trial with 0.05% of *Oregano* (*O. heracleoticum*) originated EOs showed better growth, body indices (VSI, HSI, and CF), and antioxidant property (SOD and CAT) in channel catfish (*Ictalurus punctatus*) [66]. Carvacrol and thymol are the active substances of oregano EOs; however, in this fish species, *O. vulgare* originated commercial EOs showed inferior results relative to *O. heracleoticum*. Silver catfish (*Rhamdia quelen*) was dietary administrated (2 mL/Kg) with *Aloysia triphylla* EOs [41] and bath treatment (5 and 10 mg/L) with EOs compound, eugenol [67]. Bath treatment was unable to upregulate hematological and immunological parameters, but dietary administration improved healthy blood cells (leukocyte, lymphocyte, and neutrophil) and protein levels. Most importantly, these two catfish species had increased tolerance against *A. hydrophila* infection protection after feeding or bath treatment with plant originated EOs. 

Eight weeks of feeding with *O. vulgare* EOs increased both immune and antioxidant properties and resistance against *A. hydrophila* in *Cyprinus carpio* [60,64]. EOs increased transcription levels of interleukin (IL)-1β and IL-10 and down-regulated tumor necrosis factor (TNF)-α and transforming growth factor (TGF)-β. Moreover, the increment of digestive enzyme activities and enrichment of beneficial bacterial genera in the intestinal microbial community were also found after EOs supplementation (Table 1). Feeding with *O. onites* instead of *O. vulgare*, similarly positive immunity and anti-oxidant activity modulation, and infectious disease protection was found in rainbow trout (*Oncorhynchus mykiss*) [68]. Futher, water extract of *Ocimum sanctum* leaves increased total RBC, WBC, hemoglobin, and other immune and anti-oxidant parameters in *L. rohita* [69]. 

**Table 1:** Effects of herbal essential oils on growth, immunity, and infectious diseases protection in commercial fish species.

Aquatic Species	Essential Oil	Dose and Duration	Influence	References
Mozambique tilapia (*Oreochromis mossambicus*)	Bitter lemon (*Citrus limon*)	0.5, 0.75, and 1% for 60 days	- Growth indices and feed utilization ( 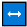 ) - Nitroblue tetrazolium (NBT), white blood cells (WBCs), Blood total protein, lysozyme, and myeloperoxidase activity (↑) - Serum glucose, cholesterol, and triglycerides (↓) - Resistance against *Edwardsiella tarda* (↑)	Baba, et al. [61]
*O. mossambicus*	Sweet orange (*C. sinensis*)	0.1, 0.3, and 0.5% for 60 days	- Growth indices and feed utilization (↑) - Lysozyme and myeloperoxidase activity, hematological and biochemical variables, i.e., hemoglobin (Hb), hematocrit (Htc), erythrocyte indices, total serum protein, albumin, and globulin (↑) - Blood glucose, cholesterol, and triglyceride (↓) - Resistance against *Streptococcus iniae* (↑)	Acar, et al. [62]
*Labeo victorianus*	*C. limon*	1, 2, 5, and 8% for 28 days	- Red blood cells (RBC), WBC, Htc, mean cell haemoglobin (MCH), haemoglobin concentration (MCHC), and neutrophils (↑) - Immunoglobulin (IgM), lysozyme activity, and respiratory burst (↑) - Resistance against *A. hydrophila* (↑)	Ngugi, et al. [63]
Nile tilapia (*O. niloticus*)	Lemongrass (*Cymbopogon citratus*) and Geranium (*Pelargonium graveolens*)	200 and 400 mg/kg for 12 weeks	- Growth indices and feed utilization (↑) - Plasma catalase; catalase (CAT), glutathione content, lysozyme activity, and total immunoglobulins; IgM (↑) - Malondialdehyde (MDA), total intestinal bacteria, coliforms, *Escherichia coli*, and *Aeromonas* spp (↓) - Resistance against *Aeromonas hydrophila* (↑)	Al-Sagheer, et al. [40]
*O. niloticus*	*Origanum vulgare*	5 and 10% for 8 weeks	- Growth indices and feed utilization (↑) - Antioxidant activities (↑) - Resistance against *Vibrio alginolyticus* (↑)	Abdel-Latif and Khalil [70]
*Tilapia zillii*	Origanum	1 g/kg for 15 days	- RBC, WBC, Hb, and differential leukocyte ( 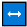 ) - Plasma proteases, antiproteases, lysozyme, and bactericidal activities (↑) - Resistance against *V. anguillarum* (↑)	Mabrok and Wahdan [65]
Channel catfish (*Ictalurus punctatus*)	*O. heracleoticum*	0.05% for 8 weeks	- Growth performance, hepatosomatic index, viscerosomatic index, and condition factor (↑) - Superoxide dismutase (SOD) and CAT (↑) - Resistance against *A. hydrophila* (↑)	Zheng, et al. [66]
Silver catfish (*Rhamdia quelen*)	*Aloysia triphylla*	2.0 mL/kg for 21 days	- Total leukocyte, lymphocyte, and neutrophil counts (↑) - Total blood protein and resistance against *A. hydrophila* (↑)	dos Santos, et al. [41]
*R. quelen*	Eugenol	Bath (5 and 10 mg/L)	- Hematological and immunological parameters ( 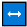 ) - Resistance against *A. hydrophila* (↑)	Sutili, et al. [67]
Common carp (*Cyprinus carpio* L.)	*O. vulgare*	0, 5, 10, 15, and 20 g/kg diet for 8 weeks	- SOD, CAT, lysozyme activity, phagocytic activity, and index (↑), and malonaldehyde (MDA) (↓) - Interleukin- (IL)-1β and IL-10 (↑) - Resistance against *A. hydrophila* (↑)	Abdel-Latif, et al. [64]
Koi carp (*C. carpio*)	*O. vulgare*	0, 500, 1500, and 4500 mg/kg for 8 weeks	- Protease, amylase, and lipase (↑) - Lysozyme, Complement C3 & C4, SOD, and glutathione peroxidase (↑) and MDA (↓) - Tumor necrosis factor (TNF)-α and Transforming growth factor (TGF)-β (↓) - *Vibrio* (↓), *Propionibacterium*, *Brevinema*, and *Corynebacterium*_1 (↑) - Resistance against *A. hydrophila* (↑)	Zhang, et al. [60]
Rainbow trout (*Oncorhynchus mykiss*)	*O. onites*	0.125, 1.5, 2.5, and 3.0 mL/kg for 90 days	- Growth indices and feed utilization (↑) - SOD, CAT, and Lysozyme activity (↑) - Resistance against *Lactococcus garvieae* (↑)	Diler, et al. [68]
*L. rohita*	*Ocimum sanctum*	0.0, 0.05, 0.1, 0.2, 0.5, and 1% for 42 days	- Superoxide anion production, lysozyme activity, plasma IgM, total serum protein, globulin, total RBC, WBC, and haemoglobin (↑) - Resistance against *A. hydrophila* (↑)	Das, et al. [69]

Variation in the treated fish compared to controls: (↑), significantly increases; (↓), significantly decreased; (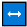), no significant change.

## 3. Essential Oils as Antiparasitic Agents

### 3.1. Acanthocephalas 

#### *Neoechinorhynchus buttnerae* 

*Neoechinorhynchus buttnerae* is an acanthocephalan parasite causing significant economic losses in *Colossoma macropomum* fish in the region of Amazon [71,72]. It was reported that *Mentha piperita*, *Lippia alba*, and *Zingiber officinale* [73] and *Piper hispidinervum*, *Piper hispidum*, *Piper marginatum*, and *Piper callosum* [74] essential oils showed 100% anthelmintic effect on *N. buttnerae*. When EO of piper hispidinervum was applied on *N. buttnerae* parasite in 0.78 mg/L concentration for 15 min, it gave the most effective result in terms of dose and time [74] (Table 2). 

### 3.2. Monogeneans 

#### 3.2.1. *Anacanthorus spathulatus*, *Notozothecium janauachensis*, and *Mymarothecium boegeri*

*Anacanthorus spathulatus*, *Notozothecium janauachensis*, and *Mymarothecium boegeri* cause significant infections in species belonging to the Serrasalmidae family as *C. macropomum* fish being in the first place [75,76]. Anthelmintic effects of *Cymbopogon citratus*, *Pterodon emarginatus*, *Lippia origanoides*, *Lippia sidoides*, and *Lippia alba* EOs on these three parasites were researched [77]. Among the EOs, the most effective one was *Lippia sidoides;* when applied as 320 mg/L for 10 min, it exhibited 100% efficacy against all three parasites [78] (Table 2). 

#### 3.2.2. *Dactylogyrus* spp. 

One of the most common parasitic pathogens in cultured freshwater fish is *Dactylogyrus* spp. [79]. Brasil, et al. [9] researched anthelmintic effects of *Lippia alba*, *Lippia origanoides*, and *Lippia sidoides* EOs on *Dactylogyrus minutus* and *Dactylogyrus extensus* parasites; and they detected that when *L. Origanoides* and *L. Sidoides* EOs were applied as 100 mg/L for 5 min, they showed 100% efficacy (Table 2). 

#### 3.2.3. *Cichlidogyrus* spp.

*Cichlidogyrus* is the parasite genus that occurs naturally in cichlid fish and has the most species among gill parasites, with its 131 different species known [80]. *Scutogyrus* species can also be dominant in the winter season among fish belonging to the Cichlidae family [81]. de Oliveira Hashimoto, et al. [82] reported that *Lippia sidoides* EO had 100% efficacy against *Cichlidogyrus* spp. and *Scutogyrus longicornis* when applied as 160 mg/L for 1 min 58 s while *Mentha piperita* EO had 100% efficacy when applied as 320 mg/L for 8 min 11 s (Table 2).

#### 3.2.4. *Dawestrema* spp.

*Dawestrema cycloancistrium* and *Dawestrema cycloancistrioides* are two of the most significant parasite types causing death and economic losses in *Arapaima gigas* fish, which are cultured in the region of Amazon [83,84]. Application of *M. piperita* EO as 160 and 320 mg/L for 30 min showed 100% efficacy on *D. cycloancistrium* and *D. cycloancistrioides* parasites [85] (Table 2). 

#### 3.2.5. *Gyrodactylus* spp.

*Gyrodactylus* spp. causes economic losses in many cultured fish species. Anthelmintic effects of *Hesperozygis ringens*, *Ocimum gratissimum*, and *Ocimum americanum* [37] and *Ocimum americanum* [86] EOs on *Gyrodactylus* spp. were researched. Only *O. americanum* EO as 50 mg/L for 1 h had the most effective anthelmintic action (98% efficacy) against *Gyrodactylus* spp. [86] (Table 2). 

### 3.3. Trepomonadea 

#### *Hexamita inflata* 

*Hexamita inflate* is a flagellated anaerobic protozoan and free-living in fresh and seawater. Moon, et al. [87] reported that *L. angustifolia* and *L. intermedia* EOs as 1 and 0.5% for 30 min exhibited 100% efficacy on *H. inflate* (Table 2).

### 3.4. Clinostomidae

#### *Euclinostomum heterostomum* 

*Euclinostomum heterostomum* is parasitic trematodes and very common in Europe, Asia, and Africa [88]. It infects muscular tissues and kidneys of freshwater fish [88,89]. *Verbesina alternifolia* and *Mentha piperita* EOs could act on *E. Heterostomum* in high doses and for a long time [90] (Table 2).

### 3.5. Oligohymenophorea

#### *Ichthyophthirius multifiliis* 

*Ichthyophthirius multifiliis* is the most famous virulent ciliated protozoan ectoparasite that invades the skin, fins, and gills of fish. de Castro Nizio, et al. [35] indicated that *Varronia curassavica* EO showed 100% efficacy against *I. multifiliis* trophont and tomont when applied as 10 mg/L and 50 mg/L for one h, respectively. *Hyptis mutabilis* (10 mg/L for 30 min) [91] and *Melaleuca alternifolia*, *Lavandula angustifolia*, and *Mentha piperita* (455 µL/L for 1 h) [92] EOs applications were also found to be effective on *I. multifiliis* (Table 2).

**Table 2:** Essential oils as antiparasitic agents.

Parasitic Pathogens	Essential Oil	Concentrations	Elimination Time/Effectiveness Concentration/Elimination Percentage	References
*Neoechinorhynchus buttnerae*	*Mentha piperita*, *Lippia alba*, and *Zingiber officinale*	360, 540, 720, 1440, and 2880 mg/L	➢ 1 h 20 min–1 h 55 min/540–2880 mg/L of *M. piperita*/100% anthelmintic ➢ 1 h 55 min/2880 mg/L of *L. alba*/100% anthelmintic ➢ 2 h 50 min/2880 mg/L of *Z. officinale*/100% anthelmintic	Costa, et al. [73]
*Neoechinorhynchus buttnerae*	*Piper hispidinervum*, *Piper hispidum*, *Piper marginatum*, and *Piper callosum*	0.19, 0.39, 0.78, 1.56, 3.125, 6.25, 12.5, 25, and 50 mg/L	➢ 15 min/0.78 mg/L of *P. hispidinervum*/100% anthelmintic ➢ 2 h/50 mg/L of *P. hispidum*/100% anthelmintic ➢ 2 h/12.5 mg/L of *P. marginatum*/100% anthelmintic ➢ 2 h/25 mg/L of *P. callosum*/100% anthelmintic	dos Santos, et al. [74]
*Anacanthorus spathulatus*, *Notozothecium janauachensis*, and *Mymarothecium boegeri*	*Cymbopogon citratus*	100, 200, 300, 400, and 500 mg/L	➢ 10 min/400 mg/L/100% anthelmintic	Gonzales, et al. [10]
*A. spathulatus*, *N. janauachensis*, and *M. boegeri*	*Pterodon emarginatus*	0, 50, 100, 200, 400, and 600 mg/L	➢ 15 min/400 and 600 mg/L/100% anthelmintic	Valentim, et al. [25]
*A. spathulatus*, *N. janauachensis*, and *M. boegeri*	*Lippia origanoides*	10, 20, 40, 80, 160, and 320 mg/L	➢ 30 min/320 and 160 mg/L/100% anthelmintic	Soares, et al. [78]
*A. spathulatus*, *N. janauachensis*, and *M. boegeri*	*L. alba*	160, 320, 640, 1280, and 2560 mg/L	➢ 20 min/1280 and 2560 mg/L/100% anthelmintic	Soares, et al. [77]
*Dactylogyrus minutus* and *Dactylogyrus extensus*	*L. alba*, *L. Origanoides*, and *L. sidoides*	10, 20, 40, 60, 80, and 100 mg/L	➢ 5 min/100 mg/L of *L. origanoides* and *L. sidoides*/100% anthelmintic	Brasil, et al. [9]
*Cichlidogyrus tilapiae*	*Ocimum gratissimum*	40, 160, and 320 mg/L	➢ 2 h/320 mg/L/100% anthelmintic	Meneses, et al. [93]
*Cichlidogyrus tilapiae*, *Cichlidogyrus thurstonae, Cichlidogyrus halli*, and *Scutogyrus longicornis*	*L. sidoides* and *Mentha piperita*	160 and 320 mg/L	➢ 1 min and 58 s/160 mg/L of *L*. *sidoides*/100% anthelmintic ➢ 8 min and 11 s/320 mg/L of *M*. *piperita*/100% anthelmintic	de Oliveira Hashimoto, et al. [82]
*Dawestrema cycloancistrium* and *Dawestrema cycloancistrioides*	*M. piperita*	80, 160, and 320 mg/L	➢ 30 min/160 and 320 mg/L/100% anthelmintic	Malheiros, et al. [85]
*Gyrodactylus* sp.	*Hesperozygis ringens* and *Ocimum gratissimum*	20 and 40 mg/L of *H. ringens* and 5 and 10 mg/L of *O. gratissimum*	➢ 1 h/10 mg/L of *O. gratissimum/50%* anthelmintic ➢ 1 h/40 mg/L of *H. ringens*/40% anthelmintic	Bandeira, et al. [37]
*Gyrodactylus* sp.	*Ocimum americanum*	10 and 50 mg/L	➢ 1h/50 mg/L/98% anthelmintic	Sutili, et al. [86]
*Ichthyophthirius multifiliis* trophonts and tomonts	*Varronia curassavica* (VCUR-001 VCUR-202 VCUR-509 VCUR-601)	10, 25, 50, 75, 100, and 200 mg/L	➢ 1 h/10 mg/L of *V. curassavica*, VCUR-202/100% antiparasitic for Trophont ➢ 1 h/50 mg/L of *V. curassavica*, VCUR-202/100% antiparasitic for Tomont	de Castro Nizio, et al. [35]
*Ichthyophthirius multifiliis*	*Hyptis mutabilis*	10 and 20 mg/L	➢ 30 min/10 mg/L/100% antiparasitic	Da Cunha, et al. [91]
*Ichthyophthirius multifiliis* trophonts	*Melaleuca alternifolia*, *Lavandula angustifolia*, and *Mentha piperita*	57, 114, 227, and 455 µL/L	➢ 1 h/455 µL/L/100% antiparasitic	Valladão, et al. [92]
*Euclinostomum heterostomum*	*Verbesina alternifolia* and *Mentha piperita*	200 to 1000 mg/L	➢ 24 h/600 mg/L of *V. alternifolia*/100% anthelmintic ➢ 24 h/1000 mg/L of *M. Piperita*/50% anthelmintic	Mahdy, et al. [90]
*Hexamita inflata*	*L. angustifolia* and *L*. × *intermedia* Miss Donnington	1, 0.5, or 0.1%	➢ 30 min/1 and 0.5%/100% antiparasitic	Moon, et al. [87]

## 4. Essential Oils as Antibacterial Agents: An In Vitro Perspective

### 4.1. Aeromonas spp.

*Aeromonas salmonicida* has been known as the causative agent of furunculosis [94]. *Aeromonas hydrophila*, *Aeromonas sobria*, and *Aeromonas veronii* are among the most common bacteria that cause motile *Aeromonas* septicemia in fish [94,95]. In addition, it is known that many different *Aeromonas* species cause disease in fish.

The antimicrobial effects of essential oils of some herbs on *Aeromonas salmonicida* subsp. *Salmonicida* has been investigated (Table 3). Hayatgheib, et al. [96] found that MIC and MBC values of essential oils (EOs) of different herbs on different *A. salmonicida* subsp. *Salmonicida* isolates were in the range of 113 to ≥3628 μg/mL, and the most effective (MIC and MBC: ≤520 μg/mL) herb species were *Cinnamomum zeylanicum/verum*, *Origanum vulgare*, *Origanum compactum*, *Origanum heracleoticum*, *Eugenia caryophyllata*, and Thymol rich *Thyme vulgaris*.

In a different study, the antimicrobial effects of *Origanum onites*, *O. vulgare*, and *Thymbra spicata* EOs on 18 different *A. salmonicida* isolates, and it was reported that EOs of these herbs formed 10 to 30 mm zone depending on the disc diffusion test, and they had moderate inhibitory depending on MIC values (800 μg/mL) [97]. Among *Thymus vulgaris*, *Laurus nobilis*, *Rosmarinus officinalis*, *Petroselinum crispum*, and *Thymus vulgaris* EOs showed the highest zone diameter with 30 mm on *A. salmonicida* [98], while *Azadirachta indica* nanoemulsion also exhibited similar results [99]. *Cinnamomum cassia* EO was reported to have a very high inhibitory effect on *A. salmonicida subsp*. with a 56 mm zone diameter [100].

Tural, et al. [98] reported that among *T. vulgaris*, *L. nobilis*, *R. officinalis*, and *P. crispum* EOs, *T. vulgaris* EO had the highest zone diameter on *Aeromonas sobria* and *Aeromonas veronii* with 31.5 mm and 36 mm, respectively. It was determined that *Origanum acutidens* EO formed a zone diameter of 32.7 mm on *Aeromonas hydrophila* [101].

*Cymbopogon nardus* [102] and *Syzygium aromaticum* [103] EOs had a strong inhibitory effect on *Aeromonas hydrophila* (ATCC 49140) and *Aeromonas* spp. with MIC values of 0.488–0.977 μg/mL and 0.015–0.031 μg/mL, respectively. It was found that *C. cassia*, *Cinnamomum aromaticum*, *Cymbopogon citratus*, and *Origanum vulgare* EOs were effective against *Aeromonas* spp., *Aeromonas salmonicida* subsp. *Salmonicida*, *A*. *hydrophila*, and *A*. *veronii* bv. *Sobria* (Mean Percent MBC: 0.02% to 0.65%) [100]. It was reported that *Mentha arvensis* and *Mentha piperita* EOs generally exhibited weak inhibitory effects on 12 different *Aeromonas* spp. Isolates (MIC > 1840 μg/mL) while *M. arvensis* EO shows moderate inhibitory (MIC: 1250 μg/mL) on only one isolate [36].

Majolo, et al. [104] investigated the antimicrobial effects of *Lippia alba*, *Lippia origanoides*, and *Lippia sidoides* EOs on *Aeromonas hydrophila* and found only the moderate inhibitory (MIC and MBC: 1250 μg/mL) effect of *L. sidoides* EO.

Among *Piper aduncum*, *Piper callosum*, *Piper hispidinervum*, *Piper hispidum*, and *Piper marginatum* EOs on 11 different *A. hydrophila* isolates, only *P. marginatum* had a strong inhibitory effect (MIC: 468.8 and 234.4 μg/mL) on three different *A. hydrophila* isolates [43].

*Ocimum gratissimum* and *Hesperozygis ringens* EOs showed a marked activity (MIC and MBC: 400 μg/mL) on *A. hydrophila*, which is among the pathogens of *Aeromonas hydrophila* and *Aeromonas veronii* (MIC and MBC: 400 μg/mL) while they exhibited a moderate inhibitory (≥800 μg/mL) on *A. veronii* [37].

A strong inhibitory effect of *Ocimum basilicum* EO with 3 μL/mL and 9 μL/mL MIC values was reported on *A. hydrophila* and *A. veronii*, respectively [105]. Among nine different herb EOs, *Conobea scoparioides* and *Lippia origanoides* EOs had remarkable activity against *A. hydrophila* with the low respective MIC and MBC values of 200 μg/mL [106]. 

It was reported that *Eucalyptus globulus*, *Lavendula angustifolia*, *Origanum vulgare*, and *Melaleuca alternifolia* nanoemulsions were more effective on *A. hydrophila* than their EOs, and among four different herbs, *O. vulgare* essential oil was found as the most effective with 25 μg/mL MIC and MBC, and the nano-emulsion was also found as the most effective with 3.12 μg/mL MIC and 12.5 μg/mL MBC [51]. However, generally moderate and weak inhibitory effects of *Ocimum americanum* [86], *Hesperozygis ringens* and *Ocimum gratissimum* [107], and *Lippia alba* [108] EOs on different *A. hydrophila* isolates were also reported. 

### 4.2. Vibrio spp., Listonella anguillarum, and Photobacterium damselae

Historically, vibrionaceae family members are the most severe infectious diseases in marine fish species [109]. The antimicrobial effects of *O. vulgare*, *M. alternifolia*, *C. citratus*, *C. verum*, and *T. vulgaris* EOs on *Vibrio campbellii*, *Vibrio harveyi*, *Vibrio vulnificus*, and *Vibrio parahaemolyticus* have been researched, and it was reported that generally moderate and weak inhibitory effects of these EOs on *Vibrio* spp [110]. Wei and Wee [102] indicated that *Cymbopogon nardus* EO showed potent inhibitory effects with 0.244 μg/mL and 0.488 μg/mL MIC values on *Vibrio* spp. and *Vibrio damsela*, respectively. Similarly, a strong inhibitory effect of *Thymus vulgaris* EO was reported, respectively, with 320 μg/mL MIC for *Vibrio ordalii* and *Vibrio anguillarum* and 80 μg/mL MIC for *Vibrio parahaemolyticus* [111]. A marked activity of *Syzygium aromaticum* EO with 0.015 μg/mL MIC values was reported on six different isolates of *Vibrio* spp. [103]. 

*O. vulgare* subsp. *Hirtum*, *O. onites*, and *O. marjorana* EOs had weak or moderate inhibitory effects on *Vibrio splendidus*, *Vibrio alginolyticus*, and *Listonella anguillarum* with zone diameter of 7.3 to 14.3 mm, 7.8 to 13.6 mm, and 9.1 to 14.1 mm, respectively [112]. It was reported that *Argania spinosa* EO had marked activity with 62.5 μL/mL MIC value on *L. Anguillarum* [113].

It was reported that *E. globulus*, *L. angustifolia*, *O. vulgare*, and *M. alternifolia* nanoemulsions were more effective on *Photobacterium damselae* than their EOs, and among these herbs, *O. vulgare* EO and nano-emulsion were found as the most effective [51].

### 4.3. Pseudomonas fluorescens 

*Pseudomonas fluorescens* is a harmful pathogen in a variety of farmed fish. It was reported that *Ocimum basilicum* EO exhibited a potent inhibitory with 9 μL/mL MIC value on *P. fluorescens* [105]. *C. Nardus* [102] and *S. aromaticum* [103] EOs showed marked activity on *Pseudomonas* spp. and *P. Aeruginosa*. *Thymus vulgaris* EO had a moderate inhibitory effect on *Pseudomonas* sp. with 640 μg/mL MIC value [111]. 

Among *T. vulgaris*, *L. nobilis*, *R. officinalis*, and *P. crispum* EOs, *T. vulgaris* EO exhibited the highest zone diameter with 26.5 mm on *P. fluorescens* [98]. *T. vulgaris* was also found as the most effective with a 13 mm zone diameter on *P. Aeruginosa* [114].

### 4.4. Citrobacter spp.

*Citrobacter* spp. is an opportunistic fish pathogen affecting farmed fish species. Bandeira, et al. [37] reported that *O. gratissimum* and *H. ringens* EOs showed a moderate or weak inhibitory (MIC and MBC: >1600 μg/mL) on *Citrobacter freundii*. Among *Achyrocline satureioides*, *Aniba parviflora*, *Aniba rosaeodora*, *Anthemis nobilis*, *Conobea scoparioides*, *Cupressus sempervirens*, *Illicium verum*, *Lippia origanoides*, and *Melaleuca alternifolia* EOs on *C. freundii*, only *L. origanoides* EO exhibited a moderate inhibitory [43]. 

It was determined that *C. freundii* showed susceptibility towards the *Argania spinosa* EO with a zone diameter of 15 mm [113], and *C. nardus* EO with a MIC value of 0.244 μg/mL [102].

### 4.5. Raoultella ornithinolytica

*Raoultella ornithinolytica* was isolated from kidneys and skin lesions of naturally diseased silver catfish (*Rhamdia quelen*), and *Ocimum gratissimum* EO showed a moderate inhibitory effect on this pathogen [37]. 

### 4.6. Nocardia seriolae

*Nocardia seriolae* is the causative agent of nocardiosis in cultured fish species [115]. Ismail and Yoshida [116] reported that MIC values of *C. Zeylanicum*, *Thymus vulgaris*, *Cymbopogon flexuosus*, and *Melaleuca alternifolia* EOs on 80 *Nocardia seriolae* isolates were in the range of 5 to >5120 μg/mL, and the most effective herb species were *C. zeylanicum* and *T. vulgaris* with MICs 5–160 μg/mL, respectively. 

### 4.7. Flavobacterium spp.

Flavobacterium species are widespread in soil habitats and fresh and marine waters and cause economic losses in cultured fish. *T. vulgaris* EO exhibited a potent inhibitory with 320 μg/mL MIC value on *F. psychrophilum* [111]. 

Previous studies have reported that *Flavobacterium* spp. showed high susceptibility towards the *S. aromaticum* EO with a MIC value of 0.031 μg/mL [103], and *C. nardus* EO with a MIC value of 0.977 μg/mL [102]. *R. officinalis* EO showed a moderate zone diameter with >~18 mm on *F. psychrophilum* [117]. A remarkable activity of *Allium tuberosum* EO with 20 μg/mL to 80 μg/mL MIC values was reported on six different isolates of *Flavobacterium columnare* [118]. 

### 4.8. Staphylococcus aureus

*Staphylococcus aureus* is an important Gram-positive opportunistic pathogen for aquaculture species. Gulec, et al. [101] reported that *O. acutidens* EO formed a zone diameter of 28 mm on *S. aureus*, *Z. officinale*, *N. Sativa*, *T. Vulgaris*, *S. Aromaticum* and *E. Sativa* EOs had no inhibitory effects on *S. aureus* [114].

### 4.9. Streptococcus spp., Lactococcus spp., and Vagococcus salmoninarum

*Streptococcaceae* family species are important Gram-positive pathogens for cultured fish. Among *L. alba*, *L. sidoides*, *M. piperita*, *O. gratissimum*, and *Z. officinale* EOs, strong inhibitory effects of *L. sidoides* EO was reported on *Streptococcus agalactiae* with 312.5 μg/mL MIC and 416.7 μg/mL MBC values [119]. It was determined that *S. agalactiae* had high susceptibility towards the *O. Basilicum* [105], *M. piperita* [45], *C. Nardus* [102], and *S. Aromaticum* [103] with MIC value of 9 μL/mL, 0.125 mg/mL, 0.244 μg/mL, and 0.015 μg/mL, respectively.

Gholipourkanani, et al. [51] determined that among *E. globulus*, *L. angustifolia*, *O. vulgare*, and *M. alternifolia* nano-emulsions and EOs, *O. vulgare* EO and/or nano-emulsion were found as the most effective on *Streptococcus iniae*. *Oliveria decumbens* EO had a zone of inhibition of 69 mm, and MIC and MBC values of 0.5 mg/mL and 2 mg/mL, respectively, on *S. iniae* [120]. 

A remarkable activity of *Z. multiflora* and *R. officinalis* EOs were reported, respectively, with 0.06 μL/mL and 0.5 μL/mL MIC, and 0.12 μL/mL and 0.25 μL/mL MBC for *S. iniae* [121]. Similarly, *R. Officinalis*, *Z. Multiflora*, *A. Graveolens*, and *E. Globulus* EOs exhibited potent inhibitory effects on *S. iniae*, and *R. Officinalis* showed the highest inhibition with a zone of 45 mm, and MIC value of 3.9 μg/mL, and MBC value of 7.8 μg/mL [122].

*Cinnamomum verum*, *Citrus hystrix*, *Cymbopogon citratus*, and *Curcuma longa* EOs had marked activity against *S. iniae* with the low respective MIC values of 40, 160, 320, and 160, respectively [123]. Pirbalouti, et al. [124] determined that *Thymus daenensis* and *Myrtus communis* EOs formed a zone diameter of 19 mm and 15.67 mm, respectively, on *S. iniae*. 

It was reported that *Streptococcus* spp. showed high susceptibility towards the *S. aromaticum* EO with a MIC value of 0.062 [103] and *C. nardus* EO with a MIC value of 0.488 [102].

*Zataria multifora*, *Thymbra spicata*, *Bunium persicum*, *Satureja bachtiarica*, and *Thymus daenensis* EOs exhibited potent inhibitory effects with MIC and MBC values ranged from 4 μL/mL to 16 μL/mL against the *L. garvieae* [125]. *Zataria multiflora*, *Cinnamomum zeylanicum*, and *Allium sativum* EOs showed a potent inhibitory (MIC: 0.12 to 0.5 µL/mL and MBC: 0.12 to 1 µL/mL) on *L. Garvieae* [126]. It was determined that *Argania spinosa* EO with a zone diameter of ~11 mm and MIC values of 125 μL/mL on *L. garvieae* [113]. 

*Thymus vulgaris* EO had marked activity with a zone diameter of 36.7 mm on *L. Garvieae* [101]. Among *T. vulgaris*, *L. nobilis*, *R. officinalis*, and *P. crispum* EOs, *T. vulgaris* EO exhibited the highest zone diameter with 29.5 mm on *L. Garvieae* [98]. 

It was found that *T. vulgaris* EO was more effective on *Lactococcus piscium* (MIC: 320 μg/mL) than *Lactococcus lactis* (MIC: 1280) and *Lactococcus lactis* subsp. *lactis* bv. *diacetylactis* (MIC: 1280) [111].

Among *Origanum vulgare, Hypericum perforatum, Rosmarinus officinalis, Zingiber officinale, Eugenia caryophyllata, Mentha piperita, Lavandula hybrid*, and *Nigella sativa* EOs, *O. vulgare* and *E. caryophyllata* EOs showed remarkable activity against Vagococcus salmoninarum with the low respective MIC values of 125 μL/mL and 250 μL/mL, respectively [42].

**Table 3:** Essential oils as antibacterial agents: an in vitro perspective.

Bacterial Pathogens	Essential Oil	Concentrations	Effective Essential Oil/Concentration/Disc/MIC/MBC/Pathogen	References
*Aeromonas salmonicida* subsp. *salmonicida* ATCC 14174	➢ *Cinnamomum zeylanicum/verum* ➢ *Origanum vulgare* ➢ *Origanum compactum* ➢ *Origanum heracleoticum* ➢ *Eugenia caryophyllata* ➢ Geraniol rich *Thyme vulgaris* ➢ Thymol rich *Thyme vulgaris* ➢ *Thymus satureoides* ➢ Thujanol rich *Thyme vulgaris* ➢ *Melaleuca alternifolia* ➢ *Cinnamomum camphora* ➢ Linalool rich *Thyme vulgaris* ➢ *Rosemary officinalis*	61 to 3628 μg/mL	➢ *C. zeylanicum/verum* MIC and MBC: 245 ➢ *O. vulgare* MIC and MBC: 226 ➢ *O. compactum* MIC and MBC: 458 ➢ *O. heracleoticum* MIC and MBC: 458 ➢ *E. caryophyllata* MIC and MBC: 520	Hayatgheib, et al. [96]
*A. salmonicida* subsp. *salmonicida* CAE 235	➢ *C. zeylanicum/verum* ➢ *O. vulgare* ➢ *O. compactum* ➢ *O. heracleoticum* ➢ *E. caryophyllata* ➢ Geraniol rich *T. vulgaris* ➢ Thymol rich *T. vulgaris* ➢ *T. satureoides* ➢ Thujanol rich *T. vulgaris* ➢ *M. alternifolia* ➢ *C. camphora* ➢ Linalool rich *T. vulgaris* ➢ *R. officinalis*	61 to 3628 μg/mL	➢ *C. zeylanicum/verum* MIC and MBC: 245 ➢ *O. vulgare* MIC and MBC: 226 ➢ *O. compactum* MIC and MBC: 458 ➢ *O. heracleoticum* MIC and MBC: 458 ➢ *E. caryophyllata* MIC and MBC: 520	Hayatgheib, et al. [96]
*A. salmonicida* subsp. *salmonicida* CAE 452	➢ *C. zeylanicum/verum* ➢ *O. vulgare* ➢ *O. compactum* ➢ *O. heracleoticum* ➢ *E. caryophyllata* ➢ Geraniol rich *T. vulgaris* ➢ Thymol rich *T. vulgaris* ➢ *T. satureoides* ➢ Thujanol rich *T. vulgaris* ➢ *M. alternifolia* ➢ *C. camphora* ➢ Linalool rich *T. vulgaris* ➢ *R. officinaliss*	61 to 3628 μg/mL	➢ *C. zeylanicum/verum* MIC and MBC: 61 ➢ *O. vulgare* MIC and MBC: 113 ➢ *O. compactum* MIC and MBC: 229 ➢ *O. heracleoticum* MIC and MBC: 458 ➢ *E. caryophyllata* MIC and MBC: 520 ➢ Thymol rich *T. vulgaris* MIC and MBC: 440	Hayatgheib, et al. [96]
*A. salmonicida* subsp. *salmonicida* CAE 258	➢ *C. zeylanicum/verum* ➢ *O. vulgare* ➢ *O. compactum* ➢ *O. heracleoticum* ➢ *E. caryophyllata* ➢ Geraniol rich *T. vulgaris* ➢ Thymol rich *T. vulgaris* ➢ *T. satureoides* ➢ Thujanol rich *T. vulgaris* ➢ *M. alternifolia* ➢ *C. camphora* ➢ Linalool rich *T. vulgaris* ➢ *R. officinalis*	61 to 3628 μg/mL	➢ *C. zeylanicum/verum* MIC and MBC: 490 ➢ *O. vulgare* MIC and MBC: 453 ➢ *O. compactum* MIC and MBC: 458 ➢ *O. heracleoticum* MIC: 458 and MBC: 916 ➢ *E. caryophyllata* MIC: 520 and MBC: 1040 ➢ Thymol rich *T. vulgaris* MIC and MBC: 440	Hayatgheib, et al. [96]
*Vibrio campbellii*	➢ *O. vulgare* ➢ *M. alternifolia* ➢ *C. citratus* ➢ *C. verum* ➢ *T. vulgaris*	50 to 3000 μg/mL	➢ *O. vulgare* MIC and MBC: 800 ➢ *M. alternifolia* MIC: 800 and MBC: 900 ➢ *C. citratus* MIC and MBC: 1500 ➢ *C. verum* MIC: 1000 and MBC: 1200 ➢ *T. vulgaris* MIC: 1900 and MBC: 2000	Domínguez-Borbor, et al. [110]
*Vibrio harveyi*	➢ *O. vulgare* ➢ *M. alternifolia* ➢ *C. citratus* ➢ *Cinnamomum verum* ➢ *Thymus vulgaris*	50 to 3000 μg/mL	➢ *O. vulgare* MIC: 700 and MBC: 800 ➢ *M. alternifolia* MIC and MBC: 800 ➢ *C. citratus* MIC: 1000 and MBC: 1100 ➢ *C. verum* MIC and MBC: 900 ➢ *T. vulgaris* MIC: 2000 and MBC: 2100	Domínguez-Borbor, et al. [110]
*Vibrio vulnificus*	➢ *O. vulgare* ➢ *M. alternifolia* ➢ *C. citratus* ➢ *C. verum* ➢ *T. vulgaris*	50 to 3000 μg/mL	➢ *O. vulgare* MIC: 900 and MBC: 1100 ➢ *M. alternifolia* MIC: 1000 and MBC: 1200 ➢ *C. citratus* MIC: 2000 and MBC: 2200 ➢ *C. verum* MIC: 1000 and MBC: 1100 ➢ *T. vulgaris* MIC and MBC: 1800	Domínguez-Borbor, et al. [110]
*Vibrio parahaemolyticus*	➢ *O. vulgare* ➢ *M. alternifolia* ➢ *C. citratus* ➢ *C. verum* ➢ *T. vulgaris*	50 to 3000 μg/mL	➢ *O. vulgare* MIC: 800 and MBC: 900 ➢ *M. alternifolia* MIC: 600 and MBC: 900 ➢ *C. citratus* MIC: 1400 and MBC: 1500 ➢ *C. verum* MIC: 1500 and MBC: 1600 ➢ *T. vulgaris* MIC and MBC: 1500	Domínguez-Borbor, et al. [110]
*Vagococcus salmoninarum*	➢ *Origanum vulgare* ➢ *Hypericu perforatum* ➢ *Rosmarinus officinalis* ➢ *Zingiber officinale* ➢ *Eugenia caryophyllata* ➢ *Menta piperita* ➢ *Lavandula hybrida* ➢ *Nigella sativa*	0.195 to 25 final well concentration for agar diffusion assay, 1000–0.01 μL/mL for MIC	➢ *O. vulgare* 17 to 20.33 mm/1.56 to 25 μL/mL/well and MIC: 125 ➢ *R. officinalis* MIC: 1000 ➢ *Z. officinale* MIC: 500 ➢ *E. caryophyllata* 17.83–18.66 mm/12.5–25 μL/mL/well and MIC 250 ➢ *M. piperita* MIC: 500 ➢ *L. hybrid* MIC: 1000 ➢ *N. sativa* MIC: >1000	Metin and Biçer [42]
*Aeromonas* spp. isolates (248, 249, 284, 351, 432, 520, 533, 561, 562, 565, 568 and 570)	➢ *Mentha arvensis* ➢ *Mentha piperita*	312.5 to 40,000 μg/mL	➢ *M. arvensis* MIC and MBC 1250 (isolate 520) ➢ *M. piperita* MIC and MBC 2500 (isolate 570) ➢ Other isolates MIC: >145sa8	Chagas, et al. [36]
*Aeromonas hydrophila* isolates (248, 249, 284, 432, 520, 533, 562, 568, 569 and 570)	➢ *Piper aduncum* ➢ *Piper callosum* ➢ *Piper* hispidinervum ➢ *Piper hispidum* ➢ *Piper marginatum*	117.2 to 30,000 μg/mL	➢ *P. marginatum* MIC: 468.8 for *A. hydrophila* (248 and 570) ➢ *P. marginatum* MIC: 234.4 for *A. hydrophila* (569) ➢ Others MIC: >937.5	Majolo, et al. [43]
*Streptococcus agalactiae*	➢ *Lippia alba* ➢ *Lippia sidoides* ➢ *Mentha piperita* ➢ *Ocimum gratissimum* ➢ *Zingiber officinale*	312 to 20,000 μg/mL	➢ *L. alba* MIC and MBC: 1666.7 ➢ *L. sidoides* MIC: 312.5 and MBC: 416.7 ➢ *M. piperita* MIC and MBC: 1250 ➢ *O. gratissimum* MIC and MBC: 2500 ➢ *Z. officinale* MIC:625 and MBC: 833.3	Majolo, et al. [119]
*Aeromonas hydrophila*	➢ *Lippia alba* ➢ *Lippia origanoides* ➢ *Lippia sidoides*	625 to 20,000 μg/mL	➢ *L. alba* MIC and MBC: 5000 ➢ *L. origanoides* MIC and MBC: 2500 ➢ *L. sidoides* MIC and MBC: 1250	Majolo, et al. [104]
*Aeromonas veronii* *Aeromonas hydrophila* *Citrobacter freundii* *Raoultella ornithinolytica*	➢ *Ocimum gratissimum*	100 to 3200 μg/mL	➢ 400 (MIC and MBC) for *Rifampicin resistant A. hydrophila* ➢ 800 (MIC) and 1600 (MBC) for *A. hydrophila and A. veronii* ➢ 1600 (MIC and MBC) for *C. freundii* ➢ 1600 (MIC and MBC) for *R. ornithinolytica*	Bandeira, et al. [37]
*A. veronii* *A. hydrophila* *C. freundii* *R. ornithinolytica*	➢ *Hesperozygis ringens*	100 to 3200 μg/mL	➢ 400 (MIC and MBC) for *Rifampicin resistant A. hydrophila* and *A. hydrophila* ➢ 1600 (MIC) and 3200 (MBC) for *C. freundii* ➢ 3200 (MIC and MBC) for *R. ornithinolytica* ➢ 800 (MIC and MBC) for *A. veronii*	Bandeira, et al. [37]
*A. hydrophila*	➢ *Achyrocline satureioides* ➢ *Aniba parviflora* ➢ *Aniba rosaeodora* ➢ *Anthemis nobilis* ➢ *Conobea scoparioides* ➢ *Cupressus sempervirens* ➢ *Illicium verum* ➢ *Lippia origanoides* ➢ *Melaleuca alternifolia*	12.5 to 6400 μg/mL	➢ *satureioides* MIC and MBC: >6400 ➢ *parviflora* MIC: 800 and MBC: 1600 ➢ *rosaeodora* MIC and MBC: 3200 ➢ *nobilis* MIC and MBC: 6400 ➢ *scoparioides* MIC and MBC: 200 ➢ *sempervirens* MIC and MBC: >6400 ➢ *verum* MIC: 1600 and MBC: 3200 ➢ *L. origanoides* MIC and MBC: 200 ➢ *M. alternifolia* MIC: 3200 and MBC: 6400	Bandeira Jr, et al. [106]
*C. freundii*	➢ *satureioides* ➢ *parviflora* ➢ *rosaeodora* ➢ *nobilis* ➢ *scoparioides* ➢ *sempervirens* ➢ *verum* ➢ *L. origanoides* ➢ *M. alternifolia*	12.5 to 6400 μg/mL	➢ *satureioides* MIC and MBC: >6400 ➢ *parviflora* MIC: 3200 and MBC: 6400 ➢ *rosaeodora* MIC and MBC: 3200 ➢ *nobilis* MIC and MBC: >6400 ➢ *scoparioides* MIC and MBC: 3200 ➢ *sempervirens* MIC and MBC: >6400 ➢ *verum* MIC and MBC: >6400 ➢ *L. origanoides* MIC and MBC: 800 ➢ *M. alternifolia* MIC and MBC: >6400	Bandeira Jr, et al. [106]
*R. ornithinolytica*	➢ *satureioides* ➢ *parviflora* ➢ *rosaeodora* ➢ *nobilis* ➢ *scoparioides* ➢ *sempervirens* ➢ *verum* ➢ *L. origanoides* ➢ *M. alternifolia*	12.5 to 6400 μg/mL	➢ *satureioides* MIC and MBC: >6400 ➢ *parviflora* MIC and MBC: 3200 ➢ *rosaeodora* MIC and MBC: 3200 ➢ *nobilis* MIC and MBC: >6400 ➢ *scoparioides* MIC and MBC: 3200 ➢ *sempervirens* MIC and MBC: >6400 ➢ *verum* MIC and MBC: >6400 ➢ *L. origanoides* MIC and MBC: 800 ➢ *M. alternifolia* MIC: 6400 and MBC > 6400	Bandeira Jr, et al. [106]
*Aeromonas hydrophila*, *Aeromonas veronii*, *Pseudomonas fluorescens*, and *Streptococcus agalactiae*	➢ *Ocimum basilicum*	3 and 6 μL/disc 3 to 300 μL/mL MIC	➢ 13.5 mm/3 μL/disc and MIC: 3 for *Aeromonas hydrophila* ➢ 22.0 mm/3 μL/disc and MIC: 9 for *Aeromonas veronii* ➢ 15.83 mm/3 μL/disc and MIC: 9 for *Pseudomonas fluorescens* ➢ 10.66 mm/3 μL/disc and MIC: 9 for *Streptococcus agalactiae*	El-Ekiaby [105]
*Streptococcus agalactiae*	➢ *Mentha piperita*	-	➢ MIC: 0.125 mg/mL	de Souza Silva, et al. [45]
*Photobacterium damselae*	➢ *Eucalyptus globulus* ➢ *Lavendula angustifolia* ➢ *Origanum vulgare* ➢ *Melaleuca alternifolia*	-	➢ *E. globulus* MIC: 25 and MBC: 50 ➢ Nano-emulsions from *E. globulus* MIC: 12.5 and MBC: 25 ➢ *L. angustifolia* MIC: 100 and MBC: 50 ➢ Nano-emulsions from *L. angustifolia* MIC: 50 and MBC: 50 ➢ *O. vulgare* MIC: 25 and MBC: 25 ➢ Nano-emulsions from *O. vulgare* MIC: 3.12 and MBC: 12.5 ➢ *M. alternifolia* MIC: 100 and MBC: 100 ➢ Nano-emulsions from *M. alternifolia* MIC: 50 and MBC: 50	Gholipourkanani, et al. [51]
*Aeromonas hydrophila*	➢ *E. globulus* ➢ *L angustifolia* ➢ *O. vulgare* ➢ *M. alternifolia*	-	➢ *E. globulus* MIC: 100 and MBC: 100 ➢ Nano-emulsions from *E. globulus* MIC: 50 and MBC: 50 ➢ *L. angustifolia* MIC: 100 and MBC: 100 ➢ Nano-emulsions from *L. angustifolia* MIC: 50 and MBC: 50 ➢ *O. vulgare* MIC: 25 and MBC: 25 ➢ Nano-emulsions from *O. vulgare* MIC: 3.12 and MBC: 12.5 ➢ *M. alternifolia* MIC: 50 and MBC: 50 ➢ Nano-emulsions from *M. alternifolia* MIC: 12.5 and MBC: 50	Gholipourkanani, et al. [51]
*Streptococcus iniae*	➢ *E. globulus* ➢ *L. angustifolia* ➢ *O. vulgare* ➢ *M. alternifolia*	-	➢ *E. globulus* MIC: 100 and MBC: 100 ➢ Nano-emulsions from *E. globulus* MIC: 100 and MBC: 100 ➢ *L. angustifolia* MIC: 100 and MBC: 100 ➢ Nano-emulsions from *L. angustifolia* MIC: 100 and MBC: 100 ➢ *O. vulgare* MIC: 25 and MBC: 25 ➢ Nano-emulsions from *O. vulgare* MIC: 3.12 and MBC: 12.5 ➢ *M. alternifolia* MIC: 100 and MBC: 100 ➢ Nano-emulsions from *M. alternifolia* MIC: 50 and MBC: 50	Gholipourkanani, et al. [51]
*Yersinia ruckeri* (2 isolates)	➢ *Thymus vulgaris* ➢ *Laurus nobilis* ➢ *Rosmarinus officinalis* ➢ *Petroselinum crispum*	15 μL/disc	➢ *T. vulgaris* 31.50 and 29.5 mm ➢ *L. nobilis* 11.5 mm ➢ *R. officinalis* 10 mm and 10.5 mm ➢ *P. crispum* 7 mm and 0 mm	Tural, et al. [98]
*Lactococcus garvieae*	➢ *T. vulgaris* ➢ *L. nobilis* ➢ *R. officinalis* ➢ *P. crispum*	15 μL/disc	➢ *T. vulgaris* 29.5 mm ➢ *L. nobilis* 18.5 mm ➢ *R. officinalis* 13 mm ➢ *P. crispum* 6 mm	Tural, et al. [98]
*Pseudomonas fluorescens*	➢ *T. vulgaris* ➢ *L. nobilis* ➢ *R. officinalis* ➢ *P. crispum*	15 μL/disc	➢ *T. vulgaris* 26.5 mm ➢ *L. nobilis* 9.5 mm ➢ *R. officinalis* 10 mm ➢ *P. crispum* 6.5 mm	Tural, et al. [98]
*Aeromonas sobria*	➢ *T. vulgaris* ➢ *L. nobilis* ➢ *R. officinalis* ➢ *P. crispum*	15 μL/disc	➢ *T. vulgaris* 31.5 mm ➢ *L. nobilis* 15 mm ➢ *R. officinalis* 17 mm ➢ *P. crispum* 7 mm	Tural, et al. [98]
*Aeromonas salmonicida*	➢ *T. vulgaris* ➢ *L. nobilis* ➢ *R. officinalis* ➢ *P. crispum*	15 μL/disc	➢ *T. vulgaris* 30 mm ➢ *L. nobilis* 13 mm ➢ *R. officinalis* 14.5 mm ➢ *P. crispum* 7.5 mm	Tural, et al. [98]
*Aeromonas veronii*	➢ *T. vulgaris* ➢ *L. nobilis* ➢ *R. officinalis* ➢ *P. crispum*	15 μL/disc	➢ *T. vulgaris* 36 mm ➢ *L. nobilis* 18.5 mm ➢ *R. officinalis* 17.5 mm ➢ *P. crispum* 7 mm	Tural, et al. [98]
*Streptococcus iniae*	➢ *Oliveria decumbens*	15 mg/disc	➢ 69 mm/disc and MIC: 0.5 mg/mL and MBC: 2 mg/mL	Vazirzadeh, et al. [120]
*Nocardia seriolae* (80 isolates)	➢ *Cinnamomum zeylanicum* ➢ *Thymus vulgaris* ➢ *Cymbopogon flexuosus* ➢ *Melaleuca alternifolia*	5 to 5120 μg/mL	➢ *C. zeylanicum* MIC: 5 to 160 ➢ *T. vulgaris* MIC: 10 to 160 ➢ *C. flexuosus* 20 to 640 ➢ *M. alternifolia* 160 to >5120	Ismail and Yoshida [116]
*Aeromonas hydrophila*	➢ *Ocimum americanum*		➢ MIC: 6400	Sutili, et al. [86]
*Yersinia ruckeri*, *Aeromonas hydrophila*, *Listonella anguillarum*, *Edwarsiella tarda*, *Citrobacter freundii* and *Lactococcus garvieae*	➢ *Argania spinosa*	0.5%, 1%, 2.5%, 5%, 7.5%, or 10% disc and 0.06 to 500 μL/mL MIC	➢ 13–18.33 mm/7.5–10%/disc and MIC: 31.25 for *Y. ruckeri* ➢ 14–17 mm/7.5–10%/disc and MIC: 62.5 for *A. hydrophila* ➢ 12.33–17 mm/7.5–10%/disc and MIC: 62.5 for *L. anguillarum* ➢ 14–17 mm/7.5–10%/disc and MIC: 125 for *E. tarda* ➢ 10–9.66 mm/7.5–10%/disc and MIC: 62.5 for *C. freundii* ➢ 11–11.33 mm/7.5–10%/disc and MIC: 125 for *L. garvieae*	Öntaş, et al. [113]
*Aeromonas salmonicida* subsp. *salmonicida*	➢ *Cinnamomum cassia* ➢ *Cinnamomum zeylanicum* ➢ *T. vulgaris* ➢ *Syzygium aromaticum* ➢ *Melaleuca alternifolia* ➢ *Rosemarinus officinalis* ➢ *Ocimum basilicum* ➢ *C. citratus* ➢ *Aniba rosaeodora* ➢ *Salvia officinalis* ➢ *Lavendula angustifolia* ➢ *O. vulgare*	25 μL of 20% solution/disc	➢ *C. cassia* 56 mm ➢ *C. zeylanicum* 27.3 mm ➢ *T. vulgaris* 42 mm ➢ *S. aromaticum* 29.3 mm ➢ *M. alternifolia* 12.7 mm ➢ *R. officinalis* 10.7 mm ➢ *O. basilicum* 6.7 mm ➢ *C. citratus* 44.7 mm ➢ *rosaeodora* 16.7 mm ➢ *S. officinalis* 12.7 mm ➢ *L. angustifolia* 12.7 mm ➢ *O. vulgare* 46 mm	Starliper, et al. [100]
*Aeromonas salmonicida* subsp. *salmonicida* (10 isolate) *Aeromonas hydrophila* (5 isolate) *Aeromonas veronii* bv. *sobria* (9 isolate) *Aeromonas caviae* *Aeromonas popoffii* (17 isolate) *Aeromonas allosaccharophila* (3 isolate) *Aeromonas encheleia* (9 isolate) *Aeromonas eucrenophila* (11 isolate) *Aeromonas molluscorum* (4 isolate)	➢ *C. cassia* ➢ *Cinnamomum aromaticum* ➢ *Cymbopogon citratus* ➢ *Origanum vulgare* ➢ *Thymus vulgaris*	Overall mean percent minimum bactericidal concentrations (MBC)	➢ *C. cassia* (Lotus): 0.02% ➢ *C*. *aromaticum*: 0.03% ➢ *C. cassia* (Aromaland): 0.04% ➢ *C. citratus* (Stony Mountain Botanicals): 0.10% ➢ *O. vulgare* (Now Foods): 0.14% ➢ *O. vulgare* (Herbal Authority): 0.16% ➢ *O. vulgare* (Stony Mountain Botanicals): 0.30% ➢ *C. citratus* (Now Foods): 0.36% ➢ *C. citratus* (Puritan’s Pride): 0.65% ➢ *T. vulgaris*, White: 2.11% ➢ *T. vulgaris*, Linalol: 2.22%	Starliper, et al. [100]
*Aeromonas hydrophila* (14 isolates)	➢ *Hesperozygis ringens* ➢ *Ocimum gratissimum*	100 to 3200 μg/mL	➢ *H. ringens* MIC and MBC: 800 to 3200 μg/mL ➢ *O. gratissimum* MIC: 200 to 1600 μg/mL ➢ and MBC: 400 to 1600 μg/mL	Sutili, et al. [107]
*A. hydrophila*	➢ *Lippia alba*	the initial concentration of 176,100 μg/mL	➢ MIC: 2862 ➢ MBC: 5998	Sutili, et al. [108]
*Lactococcus garvieae*	➢ *Zataria multiflora* ➢ *Cinnamomum zeylanicum* ➢ *Allium sativum*	1 to 0.007 μL/mL	➢ *Z. multiflora* MIC: 0.12 and MBC: 0.12 ➢ *C. zeylanicum* MIC: 0.5 and MBC: 0.5 ➢ *A. sativum* MIC: 0.5 and MBC: 1	Soltani, et al. [126]
*Streptococcus iniae* (2 isolates)	➢ *Zataria multiflora* ➢ *Rosmarinus officinalis*	1 to 0.0017 μL/mL	➢ *Z. multiflora* MIC: 0.06 and MBC: 0.5 ➢ *R. officinalis* MIC: 0.12 and 0.25 and MBC: > 1 for 2 isolates	Soltani, et al. [121]
*Staphylococcus aureus* *Lactococcus garviae* *Yersinia ruckeri* *Aeromonas hydrophila*	➢ *Origanum acutidens*	10 μL/disc	➢ 28 mm for *S. aureus* ➢ 36.7 mm for *L. garviae* ➢ 28.7 mm for *Y. ruckeri* ➢ 32.7 mm for *A. hydrophila*	Gulec, et al. [101]
*Aeromonas salmonicida*	➢ *Azadirachta indica* (Nano-emulsion)	40 μL/disc	➢ 30 mm	Thomas, et al. [99]
*Staphylococcus aureus* *Pseudomonas aeruginosa*	➢ *Z. officinale* ➢ *N. sativa* ➢ *T. vulgaris* ➢ *S. aromaticum* ➢ *E. sativa*	10 μL/disc	➢ *S. aromaticum* 4.5 mm for *S. aureus* ➢ *Z. officinale* 6.7 mm, *T. vulgaris* 13 mm, ➢ *S. aromaticum* 2 mm and *E. sativa* 10.3 mm for *P. aeruginosa*	Shehata, et al. [114]
*Edwardsiella* spp. (2 isolate) *Edwardsiella tarda* (18) Vibrio spp. (5 isolate) *Vibrio damsel* *Aeromonas* spp. (2 isolate) *Escherichia coli* (2 isolate) *Flavobacterium* spp. *Pseudomonas* spp. *Streptococcus* spp. *Aeromonas hydrophila* (ATCC 49140) *Citrobacter freundii* (ATCC 8090) *Edwardsiella tarda* (ATCC 15947) *Pseudomonas aeruginosa* (ATCC 35032), *Streptococcus agalactiae* (ATCC 13813)	➢ *Cymbopogon nardus*	-	➢ Overall mean MIC: 0.244 and/or 0.488 µg/mL ➢ *Edwardsiella* spp. (1 isolate), *E. tarda* (1 isolate), *Aeromonas* spp. (1 isolate) and *Flavobacterium* spp. MIC values: 0.977 µg/mL	Wei and Wee [102]
*Lactococcus garvieae*	➢ *Tanacetum parthenium* ➢ *Satureja bachtiarica*	100 μg/disc and 10 to 1000 μg/mL for MIC	➢ *T. parthenium* 15 mm/disc and MIC: 824 ➢ *S. bachtiarica* 25 mm/disc and MIC: 126	Fereidouni, et al. [127]
*Streptococcus iniae*	➢ *R. officinalis* ➢ *Z. multiflora* ➢ *graveolens* ➢ *E. globulus*	2 mg/disc and 7.8 to 1000 μg/mL MIC and MBC	➢ *R. officinalis* 45 mm/disc, MIC: 3.9 and MBC: 7.8 ➢ *Z. multiflora* 22 mm/disc, MIC: 62.4 and MBC: 250 ➢ *graveolens* 32 mm/disc, MIC: 7.8 and MBC: 15.6 ➢ *E. globulus* 18 mm/disc, MIC: 250 and MBC: 250	Roomiani, et al. [122]
*Listonella anguillarum*	➢ *Origanum vulgare* subsp. *hirtum* (7 different collection sample) ➢ *O. onites* (2 different collection sample) ➢ *O. marjorana*	2 μL/disc	➢ *O. vulgare* subsp. *hirtum* 9.1 to 14.1 mm ➢ *O. onites* 9.2 and 13.8 mm ➢ *O. marjorana* 11.5 mm	Stefanakis, et al. [112]
*Vibrio* *splendidus*	➢ *O. vulgare* subsp. *hirtum* (7 different collection sample) ➢ *O. onites* (2 different collection sample) ➢ *O. marjorana*	2 μL/disc	➢ O. vulgare subsp. hirtum 7.3 to 14 mm ➢ O. onites 12.6 and 14.3 mm ➢ O. marjorana 9.2 mm	Stefanakis, et al. [112]
*Vibrio alginolyticus*	➢ *O. vulgare* subsp. *hirtum* (7 different collection sample) ➢ *O. onites* (2 different collection sample) ➢ *O. marjorana*	2 μL/disc	➢ *O. vulgare* subsp. *hirtum* 7.9 to 11.5 mm ➢ *O. onites* 8.6 and 13.6 mm ➢ *O. marjorana* 7.8 mm	Stefanakis, et al. [112]
*Aeromonas salmonicida* (18 isolate)	➢ *Origanum onites* ➢ *Origanum vulgare* ➢ *Thymbra spicata* ➢ *Satureja thymbra*	20 μL/disc and 10 to 800 μg/mL for MIC	➢ *O. onites* 14 to 25 mm/disc and MIC: 800 ➢ *O. vulgare* 12 to 26 mm/disc and MIC: 800 ➢ *T. spicata* 10 to 30 mm/disc and MIC: 800 ➢ *S. thymbra* 10 to 30 mm/disc and MIC: 800	Okmen, et al. [97]
*Flavobacterium psychrophilum*	➢ *Rosmarinus officinalis*	0.0, 0.1, 0.3, 0.5, 0.7, 0.9 μL rosemary oil/μL	➢ >~18 mm, 0.1–0.9 μL rosemary oil/disc	Ostrand, et al. [117]
*L. garvieae*	➢ *Rosmarinus officinalis* ➢ *Zataria multiflora Anethum graveolens Eucalyptus globulus*	2 mg/disc and 7.8 to 1000 μg/mL MIC and MBC	➢ *R. officinalis* 24 mm/disc, MIC: 15.6 and MBC: 31.2 ➢ *Z. multiflora* 32 mm/disc, MIC: 7.8 and MBC: 15.6 ➢ *graveolens* 14.8 mm/disc, MIC: 62.4 and MBC: 125 ➢ *E. globulus* 16 mm/disc, MIC: 250 and MBC: 250	Mahmoodi, et al. [128]
*Streptococcus iniae*	➢ *Thymus daenensis* ➢ *Myrtus communis*	100 μg/disc	➢ T. daenensis 19 mm ➢ M. communis 15.67 mm	Pirbalouti, et al. [124]
*L. garvieae*	➢ *Zataria multifora* ➢ *Thymbra spicata* ➢ *Bunium persicum* ➢ *Satureja bachtiarica* ➢ *Thymus daenensis* ➢ *Myrtus communis*	4 to 1000 μL/mL for MIC and MBC	➢ *Z. multifora* MIC: 4 and MBC:8 ➢ *T. spicata* MIC: 8 and MBC:16 ➢ *B. persicum* MIC: 8 and MBC:16 ➢ *S. bachtiarica* MIC: 8 and MBC:16 ➢ *T. daenensis* MIC: 8 and MBC:16 ➢ *M. communis* MIC and MBC: >1000	Goudarzi, et al. [125]
*Lactococcus piscium* *Streptococcus phocae* *Flavobacterium psychrophilum* *Vibrio ordalii* *Vibrio anguillarum* *Vibrio parahaemolyticus* *Shewanella baltica* *Pseudomonas* sp. *Kluyvera intermedia* *Citrobacter gillenii* *Hafnia alvei* *Psychrobacter* sp. *Lactococcus lactis* *Lactococcus lactis* subsp. *lactis* bv. *diacetylactis* *Arthrobacter* sp.	➢ *Thymus vulgaris*	2.5 to 1280 μg/mL for MIC	➢ *L. piscium* MIC: 320 ➢ *S. phocae* MIC: 640 ➢ *F. psychrophilum* MIC: 320 ➢ *V. ordalii* MIC: 320 ➢ *V. anguillarum* MIC: 80 ➢ *V. parahaemolyticus* MIC: 320 ➢ *S. baltica* MIC: 640 ➢ *Pseudomonas sp*. MIC: 640 ➢ *K. intermedia* MIC: 1280 ➢ *C. gillenii* MIC: 1280 ➢ *H. alvei* MIC: 1280 ➢ *Psychrobacter* sp. MIC: 1280 ➢ *L. lactis* MIC: 1280 ➢ *L. lactis* subsp. lactis bv. ➢ *diacetylactis* MIC: 1280 ➢ *Arthrobacter* sp. MIC: 1280	Navarrete, et al. [111]
*Streptococcus iniae*	➢ *Cinnamomum verum* ➢ *Citrus hystrix* ➢ *Cymbopogon citratus* ➢ *Curcuma longa*	10 to 640 μg/mL	➢ *C. verum* MIC: 40 ➢ *C. hystrix* MIC: 160 ➢ *C. citratus* MIC: 320 ➢ *C. longa* MIC: 160	Rattanachaikunsopon and Phumkhachorn [123]
*Flavobacterium* *columnare* (6 isolate)	➢ *Allium tuberosum*	280 μg/mL	MIC: 20 to 80	Rattanachaikunsopon and Phumkhachorn [118]
*Vibrio* spp. (6 isolates) *Edwardsiella* spp. (21 isolates) *Aeromonas* spp. (2 isolates) *Escherichia coli* (2 isolates) *Flavobacterium* spp. *Streptococcus* spp. *Pseudomonas* spp. *Citrobacter freundii* (ATCC 8090), *Aeromonas hydrophila* (ATCC 49140), *Pseudomonas aeruginosa* (ATCC 35032), *Streptococcus agalactiae* (ATCC13813), *Edwardsiella tarda* (ATCC 15947)	➢ *Syzygium aromaticum*	0.015 to 0.062 μg/mL	Overall mean MIC: 0.015 to 0.062	Lee, et al. [103]

## 5. Research Gaps and Concluding Remarks

Using of herbal compounds in aquaculture is increasing day by day as a means of aquaculture sustainability. Essential oils (EOs) show beneficial effects on growth, immunity, antibacterial and antiparasitic activities in fish culture and are used as anesthetic compounds during fish handling and transportation. The efficiency of EOs depends on plant variables, chemical compositions of bioactive compounds, environmental characteristics of plant origin, and parts of plants from which EOs is extracted. Sometimes plant originated EOs possess a mixture of different compounds, which may produce undesirable side effects on fish and shellfish. Commercial pharmaceutical companies might play significant roles in refining the desirable and undesirable compounds of EOs to achieve better effects in fish culture.

Importantly, EOs molecular mechanisms for fish immunity increment, bacteria, and parasite destruction are also questionable. Future research through cell culture and in vitro identification and characterization of EOs action pathways may solve these questions. In the upcoming days, EOs optimum doses against infectious bacteria and parasites for worldwide commercial fish species should be extensively studied.

Lastly, the synergistic relationship between/among the bioactive compounds of EOs also opens a new research area. Before applying EOs in aquaculture from any new plants, local and international drug regulating agencies (FDA or EU) permission or guidelines should be needed or followed.

## Data Availability

Not applicable.
